# Supplementary material for: Correlation and mediation analysis between plasmapheresis donation behavior and bone mineral density and bone metabolism biomarkers: a cross-sectional study based on plasmapheresis donors at high risk of osteoporosis in China
Source: PeerJ. 2024 Dec 19;12:e18589. doi: 10.7717/peerj.18589 (PMC11663400; doi:10.7717/peerj.18589)
Supplement: Table S1 [file peerj-12-18589-s001.docx]

| Supplementary table 1 Comparison of ferritin and iron accumulation rates between two groups of plasma donors | | | |
| --- | --- | --- | --- |
|  | Control | Donor | *P*-value |
| Total | 276 | 277 | - |
| SF | 159.05±138.48 | 107.49±120.59 | ＜0.001 |
| Iron accumulation rate | 31.5%(87/276) | 14.1%(39/277) | ＜0.001 |
| Female | 30.6%(53/173) | 8.5%(15/177) | ＜0.001 |
| Male | 33.0%(34/103) | 24.0%(24/100) | 0.155 |
