# Supplementary material for: Correlation and mediation analysis between plasmapheresis donation behavior and bone mineral density and bone metabolism biomarkers: a cross-sectional study based on plasmapheresis donors at high risk of osteoporosis in China
Source: PeerJ. 2024 Dec 19;12:e18589. doi: 10.7717/peerj.18589 (PMC11663400; doi:10.7717/peerj.18589)
Supplement: Table S2 [file peerj-12-18589-s002.docx]

| Supplementary table 2 Correlation between plasmapheresis behavior, SF and bone metabolism markers (r) | | | | | | |
| --- | --- | --- | --- | --- | --- | --- |
| variables | total numbers | interval | recent frequency | SF | P1NP | β-CTX |
| total numbers | 1 | -0.131^*^ | 0.362^**^ | -0.306^**^ | 0.148^*^ | 0.031 |
| interval | -0.131^*^ | 1 | -0.359^**^ | 0.027 | -0.027 | -0.078 |
| recent frequency | 0.362^**^ | -0.359^**^ | 1 | -0.229^**^ | 0.096 | -0.007 |
| SF | -0.306^**^ | 0.027 | -0.229^**^ | 1 | -0.187^**^ | -0.043 |
| P1NP | 0.148^*^ | -0.027 | 0.096 | -0.187^**^ | 1 | 0.467^**^ |
| β-CTX | 0.031 | -0.078 | -0.007 | -0.043 | 0.467^**^ | 1 |

Note: ^*^*P*＜0.05，^**^*P*＜0.01，^***^*P*＜0.001.
