# Supplementary material for: Correlation and mediation analysis between plasmapheresis donation behavior and bone mineral density and bone metabolism biomarkers: a cross-sectional study based on plasmapheresis donors at high risk of osteoporosis in China
Source: PeerJ. 2024 Dec 19;12:e18589. doi: 10.7717/peerj.18589 (PMC11663400; doi:10.7717/peerj.18589)
Supplement: Table S3 [file peerj-12-18589-s003.docx]

Supplementary Table3：Multiple linear regression analysis of BMD in different sites

1. multiple regression analysis on lumbar spine L1-L4 BMD

| variable | group | Unstandardized coefficients | | Standardized coefficients | t | Sig. |
| --- | --- | --- | --- | --- | --- | --- |
|  |  | B | Std.error | Beta |  |  |
| Constant |  | 1.234 | 0.297 |  | 4.153 | 0.000 |
| Age |  | -0.009 | 0.005 | -0.132 | -1.803 | 0.073 |
| Sex | female（control） |  |  |  |  |  |
|  | male | 0.092 | 0.026 | 0.297 | 3.479 | 0.001 |
| BMI |  | 0.009 | 0.003 | 0.212 | 3.047 | 0.003 |
| Annual household income | low（control） |  |  |  |  |  |
|  | medium | -0.033 | 0.032 | -0.114 | -1.051 | 0.294 |
|  | high | -0.067 | 0.045 | -0.146 | -1.472 | 0.143 |
| Physical activity(IPAQ) | low（control） |  |  |  |  |  |
|  | medium | -0.022 | 0.024 | -0.079 | -0.949 | 0.344 |
|  | high | -0.050 | 0.098 | -0.036 | -0.508 | 0.612 |
| Protein intake | rarely（control） |  |  |  |  |  |
|  | often | 0.02 | 0.094 | 0.067 | 0.214 | 0.831 |
|  | daily | 0.022 | 0.094 | 0.075 | 0.236 | 0.814 |
| Interval |  | 0.001 | 0.001 | 0.077 | 0.922 | 0.358 |
| Total numbers |  | 1.95E-05 | 0.000 | 0.016 | 0.147 | 0.884 |
| Recent frequency |  | 0.000 | 0.001 | -0.024 | -0.213 | 0.831 |

1. multiple regression analysis on left femoral neck BMD

| variable | group | Unstandardized coefficients | | Standardized coefficients | t | Sig. |
| --- | --- | --- | --- | --- | --- | --- |
|  |  | B | Std.error | Beta |  |  |
| Constant |  | 1.308 | 0.346 |  | 3.775 | 0.000 |
| Age |  | -0.014 | 0.006 | -0.181 | -2.423 | 0.016 |
| Sex | female（control） |  |  |  |  |  |
|  | male | 0.030 | 0.031 | 0.084 | 0.956 | 0.340 |
| BMI |  | 0.011 | 0.003 | 0.226 | 3.205 | 0.002 |
| Annual household income | low（control） |  |  |  |  |  |
|  | medium | 0.020 | 0.038 | 0.060 | 0.540 | 0.590 |
|  | high | 0.004 | 0.053 | 0.007 | 0.074 | 0.941 |
| Physical activity(IPAQ) | low（control） |  |  |  |  |  |
|  | medium | -0.009 | 0.028 | -0.029 | -0.343 | 0.732 |
|  | high | 0.030 | 0.114 | 0.019 | 0.265 | 0.791 |
| Protein intake | rarely（control） |  |  |  |  |  |
|  | often | 0.050 | 0.110 | 0.143 | 0.451 | 0.652 |
|  | daily | -0.010 | 0.110 | -0.028 | -0.088 | 0.930 |
| Interval |  | -0.001 | 0.001 | -0.054 | -0.638 | 0.525 |
| Total numbers |  | -6.72E-05 | 0.000 | -0.048 | -0.434 | 0.665 |
| Recent frequency |  | 0.002 | 0.002 | 0.146 | 1.280 | 0.202 |

(3) multiple regression analysis on right femoral neck BMD

| variable | group | Unstandardized coefficients | | Standardized coefficients | t | Sig. |
| --- | --- | --- | --- | --- | --- | --- |
|  |  | B | Std.error | Beta |  |  |
| Constant |  | 1.262 | 0.376 |  | 3.358 | 0.001 |
| Age |  | -0.012 | 0.006 | -0.147 | -1.955 | 0.052 |
| Sex | female（control） |  |  |  |  |  |
|  | male | 0.090 | 0.034 | 0.232 | 2.660 | 0.009 |
| BMI |  | 0.009 | 0.004 | 0.172 | 2.415 | 0.017 |
| Annual household income | low（control） |  |  |  |  |  |
|  | medium | 0.014 | 0.041 | 0.037 | 0.338 | 0.736 |
|  | high | -0.022 | 0.058 | -0.039 | -0.378 | 0.706 |
| Physical activity(IPAQ) | low（control） |  |  |  |  |  |
|  | medium | 0.001 | 0.030 | 0.002 | 0.024 | 0.981 |
|  | high | 0.084 | 0.123 | 0.049 | 0.678 | 0.499 |
| Protein intake | rarely（control） |  |  |  |  |  |
|  | often | 0.005 | 0.119 | 0.012 | 0.039 | 0.969 |
|  | daily | -0.024 | 0.119 | -0.065 | -0.204 | 0.838 |
| Interval |  | -0.001 | 0.001 | -0.077 | -0.907 | 0.366 |
| Total numbers |  | 0.000 | 0.000 | 0.067 | 0.594 | 0.553 |
| Recent frequency |  | 0.001 | 0.002 | 0.060 | 0.519 | 0.605 |

1. multiple regression analysis on radius BMD

| variable | group | Unstandardized coefficients | | Standardized coefficients | t | Sig. |
| --- | --- | --- | --- | --- | --- | --- |
|  |  | B | Std.error | Beta |  |  |
| Constant |  | -1.308 | 1.968 |  | -0.665 | 0.507 |
| Age |  | -0.079 | 0.034 | -0.109 | -2.310 | 0.021 |
| Sex | female（control） |  |  |  |  |  |
|  | male | 2.060 | 0.187 | 0.530 | 11.039 | 0.000 |
| BMI |  | 0.060 | 0.017 | 0.159 | 3.455 | 0.001 |
| Annual household income | low（control） |  |  |  |  |  |
|  | medium | -0.092 | 0.796 | -0.024 | -0.115 | 0.908 |
|  | high | 0.125 | 0.793 | 0.032 | 0.158 | 0.875 |
| Physical activity(IPAQ) | low（control） |  |  |  |  |  |
|  | medium | 0.003 | 0.300 | 0.001 | 0.010 | 0.992 |
|  | high | -0.168 | 0.395 | -0.028 | -0.425 | 0.671 |
| Protein intake | rarely（control） |  |  |  |  |  |
|  | often | 0.603 | 0.399 | 0.156 | 1.511 | 0.132 |
|  | daily | 0.557 | 0.399 | 0.145 | 1.397 | 0.163 |
| Interval |  | -0.003 | 0.005 | -0.029 | -0.590 | 0.555 |
| Total numbers |  | 0.001 | 0.002 | 0.051 | 0.743 | 0.458 |
| Recent frequency |  | -0.013 | 0.015 | -0.067 | -0.83 | 0.407 |
