# Supplementary material for: Correlation and mediation analysis between plasmapheresis donation behavior and bone mineral density and bone metabolism biomarkers: a cross-sectional study based on plasmapheresis donors at high risk of osteoporosis in China
Source: PeerJ. 2024 Dec 19;12:e18589. doi: 10.7717/peerj.18589 (PMC11663400; doi:10.7717/peerj.18589)
Supplement: Table S4 [file peerj-12-18589-s004.docx]

Supplementary Table 4：Multiple linear regression of 25OHD

| variable | group | Unstandardized coefficients | | Standardized coefficients | Sig. |
| --- | --- | --- | --- | --- | --- |
|  |  | B | Std.error | Beta |  |
| constant |  | 23.748 | 1.772 |  | ＜0.001 |
| sex | female (control) |  |  |  |  |
|  | male | 7.214 | 0.611 | 0.433 | ＜0.001 |
| Total numbers |  | 0.021 | 0.005 | 0.239 | ＜0.001 |
| Recent frequency |  | -0.29 | 0.046 | -0.386 | ＜0.001 |
| Annual household income | low(control) |  |  |  |  |
|  | medium | -5.452 | 1.042 | -0.340 | ＜0.001 |
|  | high | -8.435 | 1.239 | -0.517 | ＜0.001 |
| Physical activity(IPAQ) | low(control) |  |  |  |  |
|  | medium | -1.142 | 0.739 | -0.064 | 0.123 |
|  | high | -3.256 | 1.28 | -0.108 | 0.011 |
| Protein intake | rarely(control) |  |  |  |  |
|  | often | -0.149 | 1.354 | -0.009 | 0.912 |
|  | daily | 3.838 | 1.355 | 0.238 | 0.005 |
| Calcium supplementation | rarely(control) |  |  |  |  |
|  | often | 1.756 | 1.384 | 0.045 | 0.205 |
