# Supplementary figures and images for: Correlation and mediation analysis between plasmapheresis donation behavior and bone mineral density and bone metabolism biomarkers: a cross-sectional study based on plasmapheresis donors at high risk of osteoporosis in China
Source: PeerJ. 2024 Dec 19;12:e18589. doi: 10.7717/peerj.18589 (PMC11663400; doi:10.7717/peerj.18589)

Supplementary Figure1：Process for inclusion and exclusion of subjects


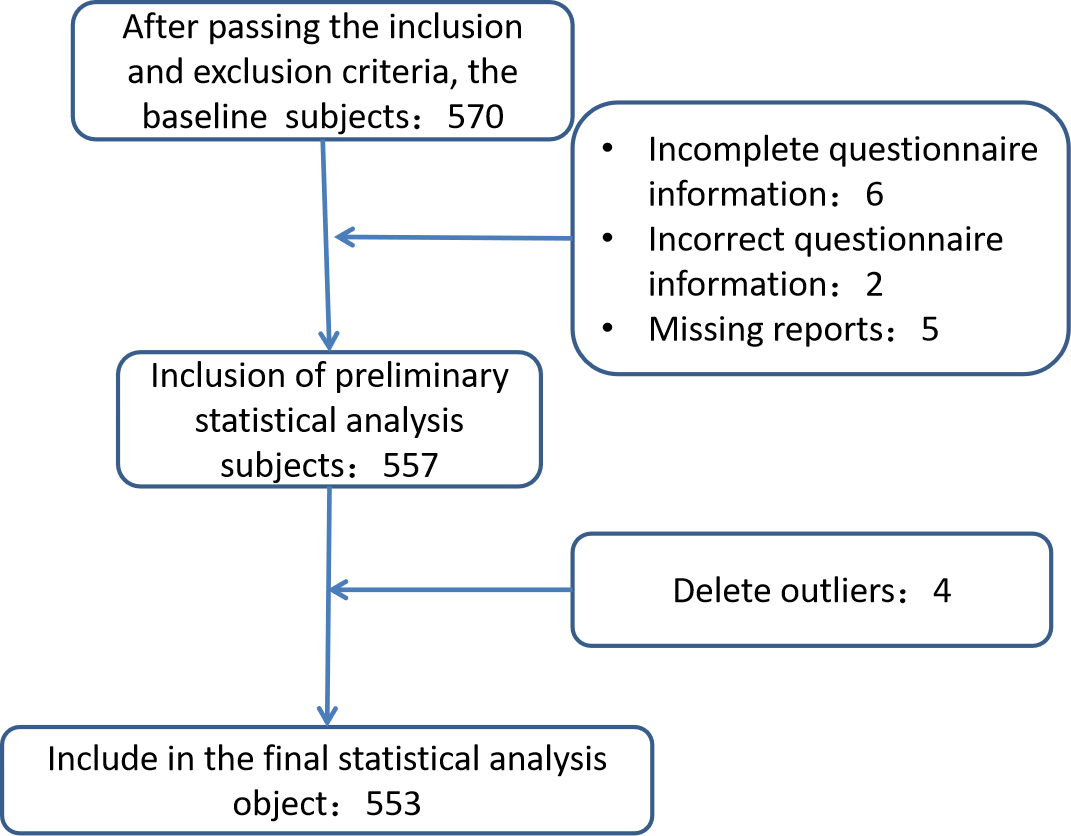

Supplement: Figure S1 [file peerj-12-18589-s005.docx]
